# NMR-Based Metabonomic Studies on Stomach Heat and Cold Syndromes and Intervention Effects of the Corresponding Formulas

**DOI:** 10.1155/2014/528396

**Published:** 2014-02-20

**Authors:** Zhongjie Zou, Bin Han, Mengjuan Gong, Shumei Wang, Shengwang Liang

**Affiliations:** School of Traditional Chinese Medicine, Guangdong Pharmaceutical University, Guangzhou 510006, China

## Abstract

Zuojin Wan (ZJW) and Lizhong Wan (LZW) have been widely used in the treatment of Stomach heat and cold syndrome (SH and SC), respectively. In this study, a proton nuclear magnetic resonance (^1^H NMR) based metabonomic approach was developed to profile SH and SC-related metabolic perturbations in rat serum and to investigate the intervention effects of ZJW and LZW on the corresponding SH and SC. Compared to the conventional macroscopic and histopathological examinations, the metabonomic approach could enable discrimination between SH and SC based on serum metabolic profiles. Meanwhile, 17 and 15 potential biomarkers associated with SH and SC, respectively, which were mainly involved in gastric dysfunction and mucosal lesions, gut microbiotal activity, transmethylation, glucose and lipid metabolism, and amino acid metabolism, were identified. Furthermore, taking the potential biomarkers as drug targets, it was revealed that administration of ZJW and LZW could exclusively reverse the pathological process of SH and SC, respectively, through partially regulating the disturbed metabolic pathways. This work showed biological basis related to SH and SC at metabolic level and offered a new paradigm for better understanding and explanation of “*Fang Zheng Dui Ying*” principle in traditional Chinese medicine from a systemic view.

## 1. Introduction

In traditional Chinese medicine (TCM), diagnosis and medication are based on “Syndrome” (“Zheng” in Chinese), which can be regarded as a profile of symptom combination [1]. Further researches using various modern methods on clarifying essence of TCM syndromes have dramatically increased in number in recent years [2–7]. Additionally, in order to obtain maximal therapeutic effects and to reduce the risk of unexpected adverse reactions, a formula must be prescribed to patients with a complementary TCM syndrome [8] (theory of “*Fang Zheng Dui Ying*,” translated as “formula corresponding to specific syndrome”). Consequently, in-depth understanding of *Fang Zheng Dui Ying *principle through modern research will greatly facilitate rational and safe clinical use of Chinese herbal medicines and will have enormous implications for personalized health care.

According to the differentiation theory in TCM, stomach heat syndrome (SH) is characterized by scorching pain in epigastric region, acid regurgitation, thirst, preference for cold drinks, polyorexia, constipation, scanty yellowish urine, reddish tongue with yellow fur, slippery and rapid pulse, halitosis, and so forth, while stomach cold syndrome (SC) is featured by dull pain in the epigastric region which is aggravated by cold foods or temperatures and alleviated by warm foods or temperatures, bland taste in the mouth without thirst, loose stool, light-colored tongue with whitish slippery fur, tense and slow pulse, dispiritedness and lassitude, excessive saliva, and so forth [9]. Symptoms of both syndromes are usually seen in acute and chronic gastritis [10, 11], digestive ulcer [12], stomach cancer [13], and so forth. Although several biochemical markers, such as thromboxane B_2_ (TXB_2_), 6-keto-prostaglandin F_1*α*_ (6-keto-PGF_1*α*_), tumor necrosis factor-*α* (TNF-*α*), interleukin-2 (IL-2), interleukin-8 (IL-8), and prostaglandin E_2_ (PGE_2_) [14–16], have been identified in SH and SC, the definite molecular pathogenesis of both syndromes still remains unclear. A recent study using network-based systems biology approach revealed that with leptin as a biomarker, gastritis patients with cold syndrome experienced low levels of energy metabolism, while the CCL2/MCP1 biomarker indicated that immune regulation was intensified in heat syndrome patients [17]. Moreover, syndrome differentiation in TCM relies much more on the experience of TCM practitioner and the patient's expression, indicating that it is particularly urgent to develop accurate and objective methods for diagnosis of syndromes and evaluation of efficacy of TCM treatment. Research on integrating next-generation sequencing and traditional tongue diagnosis illustrated the potential of the tongue-coating microbiome as a novel holistic biomarker for characterizing patient subtypes [18].

Zuojin Wan (ZJW) [16], a famous prescription with a long history of clinical use for relieving symptoms caused by SH, is comprised of two Chinese medicines: Rhizoma Coptidis and Fructus Euodiae. Lizhong Wan (LZW) [19], prescribed to patients with SC, is composed of four Chinese medicines: Radix Ginseng, Rhizoma Zingiberis, Rhizoma Atractylodis Macrocephalae, and Radix Glycyrrhizae. Experimental verification of the effectiveness of ZJW and LZW in the treatment of the corresponding SH and SC will be beneficial for the rational clinical use of both formulas.

Fortunately, as one of the latest and most exciting “-omic” sciences, metabonomics adopts a “top-down” strategy to reflect the function of organisms from the terminal symptoms of the metabolic network and to understand metabolic changes of a complete system caused by interventions in a holistic context [20]. This property agrees with the holistic thinking of TCM, suggesting that metabonomics has the potential to impact our understanding of Chinese medicine theory and plays a critical role on the modern research of TCM. Metabonomics has now become a versatile tool to achieve a comprehensive evaluation of efficacy, safety, and action mechanisms of Chinese medicines, as well as to promote the modernized study of TCM syndromes [21, 22].

In metabonomic studies, nuclear magnetic resonance (NMR) spectroscopy has been extensively used to explore the metabolic profiling of biofluids [23–26], due to its high reproducibility, nondestructiveness, nonselectivity in metabolite detection, and the ability to provide detailed information on molecular structures [27, 28]. In order to uncover the latent biochemical information that are of diagnostic or other classification value from the complex spectral data, multivariate statistical analyses such as principal component analysis (PCA) and partial least-squares discriminant analysis (PLS-DA) are generally performed [29].

In the present study, we employed a NMR-based metabonomic platform in conjunction with multivariate statistical analysis to probe metabolic perturbations in rats serum induced by SH and SC and to investigate the intervention effects of ZJW and LZW. The results demonstrated biological basis at metabolic level to enable discrimination between SH and SC and provided novel insights into understanding and explanation of *Fang Zheng Dui Ying *principle at functional level.

## 2. Materials and Methods

### 2.1. Chemicals and Reagents

Rhizoma Coptidis, Fructus Euodiae, Radix Ginseng, Rhizoma Zingiberis, Rhizoma Atractylodis Macrocephalae, Radix Glycyrrhizae, and Fructus Capsici were purchased from Tongrentang Group Co., Ltd. (Beijing, China) and authenticated by Professor Bin Han (School of Traditional Chinese Medicine, Guangdong Pharmaceutical University, China).

Distilled water was purified using a Milli-Q ultrapure water system (Millipore, Bedford, MA, USA). Deuterium oxide (D_2_O, 99.9%) was purchased from Sigma-Aldrich (St. Louis, MO, USA). Sodium dihydrogen phosphate dihydrate (NaH_2_PO_4_·2H_2_O), disodium hydrogen phosphate dodecahydrate (Na_2_HPO_4_·12H_2_O), sodium hydroxide (NaOH), and absolute ethanol were obtained from Guangzhou Chemical Reagent Factory (Guangdong, China).

### 2.2. Preparation of the Decoctions of ZJW and LZW

According to the original composition recorded in the literature [16, 19], the decoctions of ZJW and LZW were prepared using the following procedures. The crude drugs of Rhizoma Coptidis 18 g and Fructus Euodiae 3 g were extracted two times under thermal reflux with 210 mL of ultrapure water for 1.5 h each time. After filtration, the extract was combined and concentrated under reduced pressure. Finally, the decoction of ZJW was made at a concentration of 0.14 g/mL (expressed as the weight of raw materials). All the raw materials, including 15 g of Radix Ginseng, 15 g of Rhizoma Zingiberis, 15 g of Rhizoma Atractylodis Macrocephalae, and 15 g of Radix Glycyrrhizae were refluxed two times with 600 mL of ultrapure water for 1.5 h each time. The filtrate was merged and evaporated on a Buchi rotary evaporator to give the decoction of LZW with final concentration of crude drugs at 0.36 g/mL.

### 2.3. Preparation of the Suspension of Fructus Capsici and NaOH Solution

1 L of ultrapure water was mixed with dried material (80 g) of Fructus Capsici, which was pulverized finely and passed through an 120 mesh sieve, to obtain the suspension of Fructus Capsici (80 mg/mL). NaOH (12 g) was dissolved in 1 L of ultrapure water to prepare 0.3 mol/L NaOH solution.

### 2.4. Animal Handling and Sample Collection

The protocol of the study was approved by the Ethics Committee of Guangdong Pharmaceutical University, China. The investigation was conducted in accordance with the ethical principles of animal use and care.

A total of 42-male Sprague-Dawley (SD) rats (180 ± 10 g, license no. SCXK 2011-0029) were purchased from the Experimental Animal Center of Sun Yat-Sen University (Guangdong, China). All rats were kept in plastic cages at a barrier system with regulated temperature (22 ± 2°C) and humidity (50  ±  10%), and on a 12 h dark/light cycle with lights on at 8:00 am. Food and tap water were provided *ad libitum*. After 3 days of acclimation, the animals were transferred to individual metabolic cages and randomly divided into seven groups with six rats in each group as follows: (1) Control group in which ultrapure water was administrated orally via gavage at about 2 mL twice daily (at 9:00 am and 15:00 pm) for consecutive 6 days and once on day 7; (2) SH model group in which the suspension of Fructus Capsici was administrated orally at the dose of 10 mL/kg body weight twice daily for consecutive 6 days followed by oral administration of absolute ethanol (1 mL) once on day 7 after rats fasted and were allowed free access to water for 12 h [30]; (3) SH + ZJW/LZW treatment group which received the suspension of Fructus Capsici and absolute ethanol in the same manner as SH model group and was treated with the decoction of ZJW (1.4 g/kg body weight) or LZW (3.6 g/kg body weight) by gastric instillation once a day from day 4 to day 7 (1 h prior to the administration of suspension of Fructus Capsici and absolute ethanol), respectively; (4) SC model group in which 4°C cold ultrapure water was administrated orally at the dose of 10 mL/kg body weight twice daily for consecutive 6 days followed by oral administration of 0.3 mol/L NaOH solution (1 mL) once on day 7 after rats fasted and were allowed free access to water for 12 h [30]; (5) SC + ZJW/LZW treatment group which received the 4°C cold ultrapure water and NaOH solution in the same manner as SC model group and was treated with the decoction of ZJW (1.4 g/kg body weight) or LZW (3.6 g/kg body weight) by gastric instillation once a day from day 4 to day 7 (1 h prior to the administration of 4°C cold ultrapure water and NaOH solution), respectively. The dose level in this study was set according to the literature [16, 19]. Three hours after the last dosing on day 7, blood was collected from the retroorbital plexus after rats were anaesthetized with diethyl ether inhalation and allowed to clot for 1 h at 4°C, and then centrifuged at 3500 g for 15 min at 4°C to remove any precipitates. The resulting serum samples were stored at −80°C for further analysis. All experimental rats were sacrificed following blood collection. The stomach was rapidly removed and opened along the greater curvature, and then examined for lesions.

### 2.5. Gross Evaluation of Gastric Lesions

Following the harvest, the stomach was immediately flushed with saline. Gastric ulcer on the gastric mucosa appears as elongated bands of hemorrhagic lesions. The total ulcer area (UA) of each stomach was measured by planimetry (mm^2^) under a dissecting microscope (1.8x) [31]. The percentage of inhibition was calculated using the following formula:

(1) $$\begin{array}{c} \text{Inhibition}\left(\%\right)=\frac{\left[\left(\text{UA}\;\text{model}-\text{UA}\;\text{treatment}\right)\right]}{\text{UA}\;\text{model}}\times 100\%. \end{array}$$

The data were expressed as mean ± standard deviation (S.D.). Statistical significance was determined by one-way analysis of variance (ANOVA) followed by Tukey's post hoc test using SPSS 20.0 (SPSS Inc., Chicago, IL, USA). The levels of significance were set as *P* < 0.05.

### 2.6. Histopathology

A small fragment of the gastric tissue from each animal was fixed in 10% buffered formalin solution and embedded in paraffin wax. Sections 5 *μ*m in thickness were made and stained with hematoxylin and eosin (H&E) for histopathological examination under a light microscope by an experienced histologist who was blinded to the treatment regimen.

### 2.7. Sample Preparation

After serum samples were thawed at room temperature, 50 *μ*L of buffer solution (0.2 mol/L Na_2_HPO_4_ and 0.2 mol/L NaH_2_PO_4_, pH7.4) and 50 *μ*L of D_2_O were mixed with 400 *μ*L of each serum sample in a 5 mm NMR tube. The D_2_O provided a field-frequency lock solvent for the NMR spectrometer.

### 2.8. ^1^H NMR Spectroscopic Analysis

^1^H NMR spectra of the serum samples were randomly measured at 298 K on a Bruker AVANCE III 500 MHz spectrometer (BrukerBiospin, Rheinstetten, Germany) operating at 500.13 MHz ^1^H frequency by using the water-suppressed Carr-Purcell-Meiboom-Gill (CPMG) spin-echo pulse sequence (RD-90°-(*τ*-180°-*τ*)*n*-ACQ) with a total spin-echo delay (2*nτ*) of 100 ms to attenuate broad signals from proteins and lipoproteins. Typically, 128 free induction decays (FIDs) were collected into 32 k data points over a spectral width of 10,000 Hz with a relaxation delay of 3 s and an acquisition time of 3.28 s. The FIDs were zero-filled to double size and weighted by an exponential function with a 0.3 Hz line-broadening factor prior to Fourier transformation.

### 2.9. Data Processing and Analysis

The acquired NMR spectra were manually corrected for phase and baseline distortions, referenced to methyl group of lactate (*δ* 1.33), and segmented into regions of equal width (0.01 ppm) in the range of *δ* 0.5–9.5 using MestReNova 6.1 software package (Mestrelab Research S.L, Santiago de Compostela, Spain). The regions containing residual water (*δ* 4.68–5.22), ethanol (*δ* 1.14–1.22 and 3.62–3.70), and its metabolite acetate (*δ* 1.91–1.93) were excluded. The integral values of the remaining regions were then normalized, within each sample, to the sum of all integrals in that sample to reduce any significant concentration differences between samples and then multiplied by 10,000. The resultant integral data were imported into SIMCA-P 12.0 software (Umetrics, Umea, Sweden) for multivariate analysis after mean-centering and pareto scaling, a technique that increased the importance of low abundance ions without significant amplification of noise.

Principal component analysis (PCA), used to detect intrinsic clusters and outliers within the data set, was followed by partial least-squares discriminant analysis (PLS-DA) to achieve the maximum separation between samples and identify differential metabolites that account for the separation between groups. To avoid overfitting of PLS-DA models, a default 7-fold cross-validation method was applied, from which values of goodness of fit (*R* ^2^ *Y*) and predictability (*Q* ^2^) were computed. In addition, model validation was also performed by 999 times permutation tests. Metabolites with VIP (variable importance in the projection) values ≥ 1.0 were considered significant in this study. In parallel, univariate statistical analysis with the critical *P* value of 0.05 was performed using SPSS 20.0 (SPSS Inc., Chicago, IL, USA) to validate those major contributing variables from the PLS-DA models. Only those metabolites that meet the two criteria are eventually considered as potential biomarkers. The heat map of relative levels of differential metabolites was plotted using heatmap.2 package in R3.0 environment downloaded freely from the Web site (http://www.r-project.org/).

## 3. Results and Discussion

In TCM theory, SH is usually caused by excessive intake of pungent and warm foods, such as Fructus Capsici, which transforms into heat and fire, or by emotional upsets and stagnation of *qi* which transforms into fire and attacks the stomach, whereas SC is mainly induced by excessive ingestion of cold and uncooked foods, such as cold water, or by cold attacking on the epigastrium and abdomen, or by overstrain [9]. Previous studies showed that heat-related (e.g., Fructus Capsici and ethanol) [14, 16, 19] and cold-related (e.g., cold water and NaOH) [15, 16, 19] pathogenic factors could be used to induce various pathological alterations in experimental animals that resembled those observed in patients with SH and SC, respectively.

### 3.1. Gross Gastric Lesions Evaluation and Histopathological Examinations

**Figure 1:**
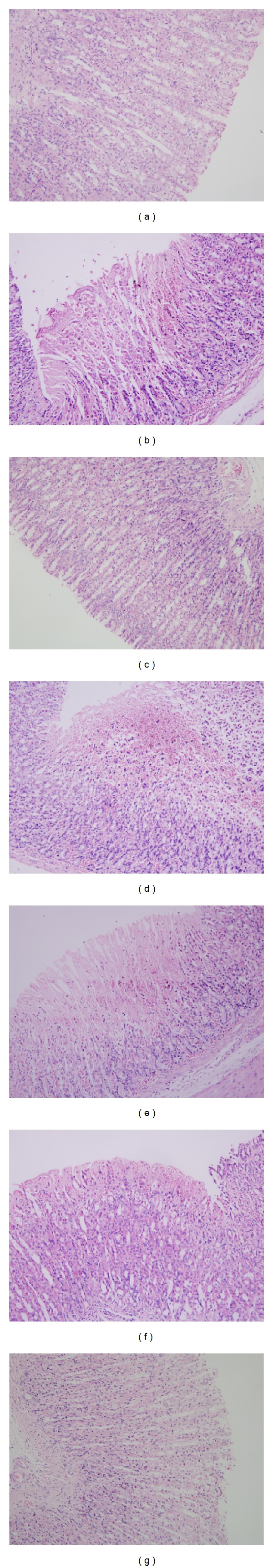
Photomicrographs of H&E-stained gastric mucosa from rats (magnification ×200). (a) Control group, (b) SH model group, (c) SH + ZJW treatment group, (d) SH + LZW treatment group, (e) SC model group, (f) SC + ZJW treatment group, and (g) SC + LZW treatment group.

Macroscopic examination of the gastric mucosa showed that SH and SC model groups had gastric mucosal injuries such as hemorrhage and hyperemia, whereas no abnormalities or lesions were found in the normal control group. Conversely, SH + ZJW and SC + LZW treatment groups had attenuated gastric lesions when compared with SH and SC model groups, respectively, as evidenced by the reduction of ulcer area in both groups (Table 1). Administration of ZJW and LZW to the corresponding SH and SC model group reduced the ulcer area formation by 95.20% and 92.99%, respectively. However, SH + LZW and SC + ZJW treatment groups had similar gastric mucosal injuries as SH and SC model groups. In the histopathological examinations, severe disruption to the superficial region of the gastric gland with epithelial cell loss and intense hemorrhage was observed in SH and SC model groups, but only the SH + ZJW and SC + LZW treatment groups experienced reduced gastric damage (Figure 1). These findings are consistent with results of previous studies [14–16, 19] which proved that the gastric lesions induced by SH and SC in rats could only be alleviated by administration of ZJW and LZW, respectively. However, from Table 1 and Figure 1, no significant difference between SH and SC model groups was observed and this indicated that macroscopic and histopathological analyses were not suitable for differentiating between SH and SC and that more sensitive methods should be applied.

### 3.2. Analysis of ^1^H NMR Spectroscopic Profiles of Rat Serum

**Figure 2:**
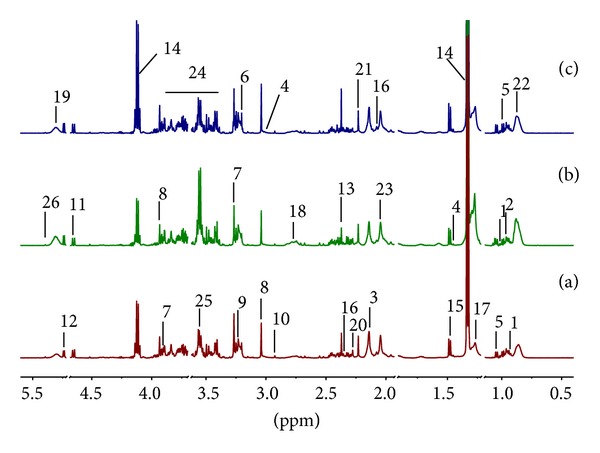
Representative ^1^H NMR spectra of rat serum. (a) Control group, (b) SH model group, and (c) SC model group. Keys: 1: Isoleucine; 2: Leucine; 3: Methionine; 4: Lysine; 5: Valine; 6: Choline; 7: Betaine; 8: Creatine; 9: Phosphocholine; 10: Trimethylamine; 11: *β*-Glucose; 12: *α*-Glucose; 13: Pyruvate; 14: Lactate; 15: Alanine; 16: Glutamate; 17: very low-density/low-density lipoprotein (VLDL/LDL) –CH_2_–; 18: Lipid =CHCH_2_CH=; 19: Lipid CH=CH; 20: Acetoacetate; 21: Acetone; 22: VLDL/LDL –CH_3_–; 23: N-acetyl signals from glycoproteins; 24: Glucose and amino acid CH; 25: glycine; 26: Allantoin.

Representative ^1^H NMR spectra of rat serum were shown in Figures 2 and 1S in Supplementary Material available online at http://dx.doi.org/10.1155/2014/528396. A CPMG pulse sequence was used to emphasize the small metabolites in serum by attenuating the resonances from macromolecules such as lipoproteins [27]. The assignment of the resonances to specific metabolites was achieved based on matching the acquired NMR data (i.e., chemical shifts, coupling constant, and multiplicity) to the reference spectra in the Human Metabolome Database version 3.0 [32], as well as other existing databases and previous reports [23, 25, 33, 34]. The mostly identified endogenous metabolites were labeled in the spectra. The high interindividual variability in serum profiles and their large complexity make any attempt of visual comparison of these spectra an unproductive task. Instead, multivariate data analysis allows finding consistent variation patterns within the data set.

### 3.3. Metabolic Changes in Rat Serum Induced by SH and SC

**Figure 3:**
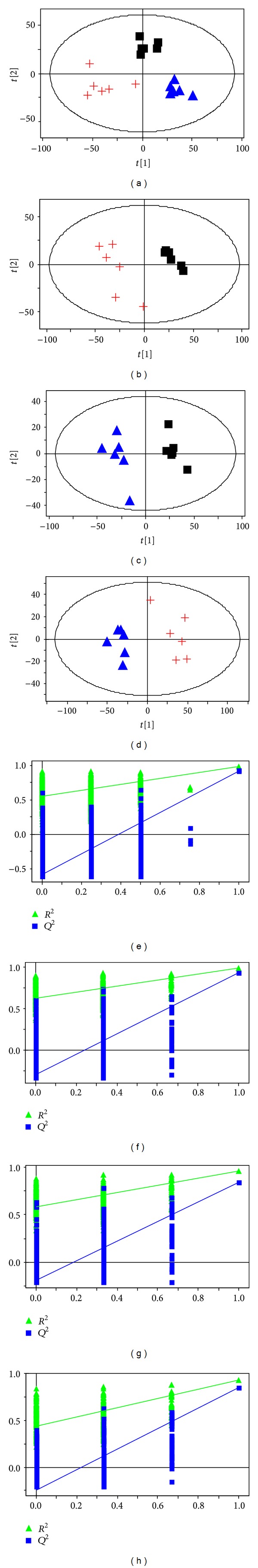
Results of multivariate data analysis and validation based on ^1^H NMR spectra of rat serum samples. (a–d) PLS-DA scores plots and (e–h) corresponding plots of permutation tests. (black square) Control group, (red plus) SH model group, and (blue triangle) SC model group. Permutation tests were carried out with 999 random permutations in PLS-DA models.

PCA was first performed to get an overview of the difference of serum metabolite profiles in Control, SH, and SC groups and no outlier was observed (Figure 2(S)). In order to maximize the separation between different groups and to obtain information on the metabolites significantly contributing to classifications, the PLS-DA models were subsequently constructed (Figure 3). The SH and SC model groups were clearly separated from the Control group (Figures 3(a), 3(b), and 3(c)) suggesting that serum metabolic profiles of rats with SH and SC were significantly changed compared with those of healthy controls. In the meanwhile, a good separation was also achieved between the SH and SC groups (Figures 3(a) and 3(d)) indicating obvious metabolic difference between the two syndromes. The model parameters were as follows: *R* ^2^ *Y* = 0.98, *Q* ^2^ = 0.91 for Figure 3(a); *R* ^2^ *Y* = 0.99, *Q* ^2^ = 0.94 for Figure 3(b); *R* ^2^ *Y* = 0.96, *Q* ^2^ = 0.84 for Figure 3(c); *R* ^2^ *Y* = 0.93, and *Q* ^2^ = 0.85 for Figure 3(d). In general, excellent models were obtained when values of *R* ^2^ *Y* and *Q* ^2^ were above 0.8 [35]. Furthermore, the robustness of these PLS-DA classification models was assessed by 999 times permutation tests. The *R* ^2^ and *Q* ^2^ values derived from the permuted data were lower than the original ones, and all the blue regression lines of the *Q* ^2^-points intersected the vertical axis below zero, suggesting the validation of these PLS-DA models (Figures 3(e)–3(h)) [36].

The differentiation between SH and SC could not be made on the basis of macroscopic and histopathological examinations as mentioned above, but the metabonomic approach applied in this study was capable of distinguishing SH from SC, suggesting that metabonomics might play a crucial role in the modern study of TCM syndromes.

### 3.4. Identification of the Differential Endogenous Metabolites Associated with SH and SC

**Figure 4:**
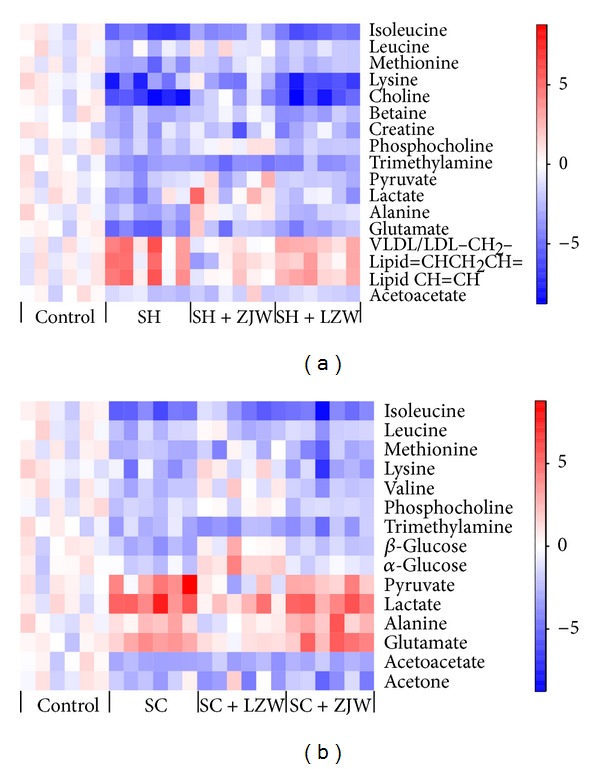
Heat map of the differential metabolites from rat serum in different groups. (a) SH and (b) SC. Each metabolite is represented by a single row of colored boxes, whereas columns correspond to different samples. Every concentration value was standardized according to the control group, that is, by subtracting mean and dividing by the standard deviation of healthy controls. In that way, the metabolites concentrations were expressed in values of standard deviation from the control group. Red and blue colors represent elevation or reduction of a given metabolite concentration, respectively.

Selected according to the VIP values from the PLS-DA models (VIP ≥ 1) and the *P* values from univariate statistical analysis (*P* < 0.05), 17 and 15 endogenous metabolites associated with SH and SC, respectively, were identified as potential biomarkers (Table 2). To visualize the alterations of the differential metabolites, a heat map was generated based on their relative levels (Figure 4). Among the differential metabolites identified in SH and SC, 11 metabolites were altered in both syndromes with 10 only in one. Decreased levels of isoleucine, leucine, methionine, lysine, phosphocholine, trimethylamine, and acetoacetate were observed in SH and SC groups. Pyruvate, lactate, alanine, and glutamate were down-regulated in SH group while up-regulated in SC group. The SH group had higher levels of VLDL/LDL –CH_2_–, lipid =CHCH_2_CH=, and lipid CH=CH, and lower levels of choline, betaine, and creatine, whereas the SC group had reduced levels of valine, *β*-glucose, *α*-glucose, and acetone.

### 3.5. Biological Explanation for Potential Biomarkers

The stomach is a major protein-digesting organ between the esophagus and the small intestine. It stores swallowed food, mixes the food with stomach acids, and then sends the mixture on to the small intestine. Previous studies [19] revealed that SH and SC in rats induced a decrease of food consumption and weight loss, possibly due to indigestion and stomach discomfort such as scorching or dull pain. In the present study, the metabolite profiling of serum showed that in both syndromes, 5 essential amino acids including isoleucine, leucine, methionine, lysine, and valine, which only could be obtained from the diet, were found significantly decreased, indicating gastric dysfunction induced by SH and SC.

**Figure 5:**
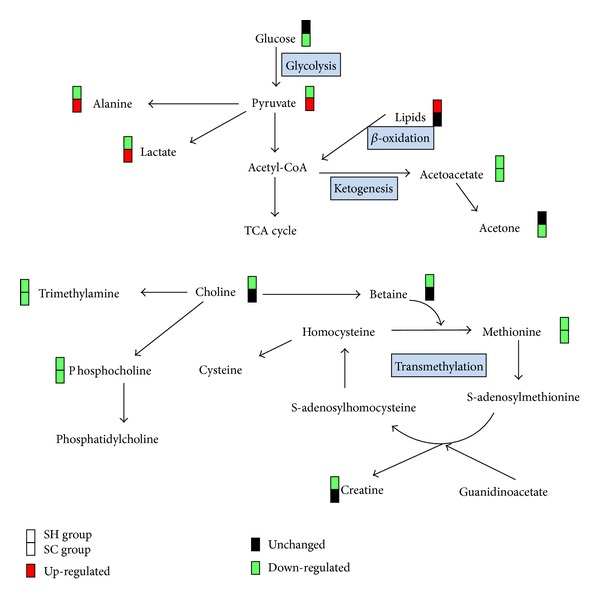
Schematic representation of the most disrupted metabolic pathways. The red squares represent significant up-regulations of metabolites in SH or SC model group compared with control group, whereas the green squares indicate down-regulations. The black ones depict there is no significant change.

Phosphatidylcholine (lecithin) is a key building block of cell membrane bilayers and is now receiving increasing attention as protective agent in the gastrointestinal barrier [37]. Phosphocholine, an important intermediate in the synthesis of phosphatidylcholine in tissues, is derived from the conversion of choline catalyzed by choline kinase. The detected lower level of phosphocholine induced by SH and SC and decreased concentration of choline induced by SH (Figure 5) possibly led to the gastric mucosal lesions in both syndromes as observed in macroscopic and histopathological examinations. In the current investigation, the reduced level of trimethylamine (TMA) was observed in serum in rats with SH and SC. Several strains of gut bacteria have been shown to decompose choline to TMA [38]; therefore, it was plausible to suggest that SH and SC caused a disturbance in gut microbiotal colonies in rats. In addition, betaine, methionine, and creatine were significantly down-regulated in SH model group, implying a disruption in transmethylation. Choline is a major source for methyl groups via one of its metabolites, betaine, that participates in the S-adenosylmethionine (SAM) synthesis pathways [39]. And it is well known that creatine, as byproduct of choline metabolism, is formed when guanidinoacetate (GAA) receives the SAM methyl under the action of guanidinoacetate methyltransferase [40]. So it was reasonable to expect that SH but not SC induced changes in transmethylation. Altogether, among the major pathways utilizing choline (Figure 5), all the three seemed to be interfered by SH, but only two by SC.

Changes in a number of metabolites involved in energy metabolism were observed in this work. SH model group showed marked depletion in serum concentration of pyruvate and lactate together with a rising trend in glucose level (Table 2), whereas SC model group showed a drop in serum glucose and elevated levels of pyruvate and lactate. Pyruvate, the product of glycolysis, represents an important junction point in carbohydrate catabolism under aerobic and anaerobic conditions, and lactate is the end-product of glucose metabolism under anaerobic conditions. Although changes of the tricarboxylic acid (TCA) cycle intermediates in glucose metabolism under aerobic conditions were not detected in this study, it was conceivable to suggest that slowdown and acceleration of glycolytic activity were induced by SH and SC, respectively. Furthermore, NMR spectroscopy measurements on rat serum highlighted an increase in lipids and a decrease in acetoacetate in SH, while a decrease in acetoacetate and acetone was accompanied by a decreasing trend of lipids concentrations (Table 2) in SC. These findings revealed the suppression of lipid *β*-oxidation in SH and ketogenesis in both syndromes in energy production. Based on the results described above, we could deduce that in order to keep the body in balance, energy metabolism in rats with stomach heat and cold state moved in completely opposite directions except ketogenesis.

Alanine, the *α*-amino acid analog of the *α*-keto acid pyruvate, is most commonly produced by the reductive amination of pyruvate via alanine transaminase. This reversible reaction involves the interconversion of alanine and pyruvate, coupled to the interconversion of *α*-ketoglutarate and glutamate [41]. Thus, the altered levels of pyruvate, alanine, and glutamate (Figure 5) might reflect disorders of transamination and could be used as an index of disturbance in amino acid metabolism caused by SH and SC. And the contrary change trends of the three metabolites in the two syndromes implied inhibition and stimulation of amino acid metabolism in SH and SC, respectively.

### 3.6. Intervention Effects of ZJW and LZW on the Corresponding SH and SC

**Figure 6:**
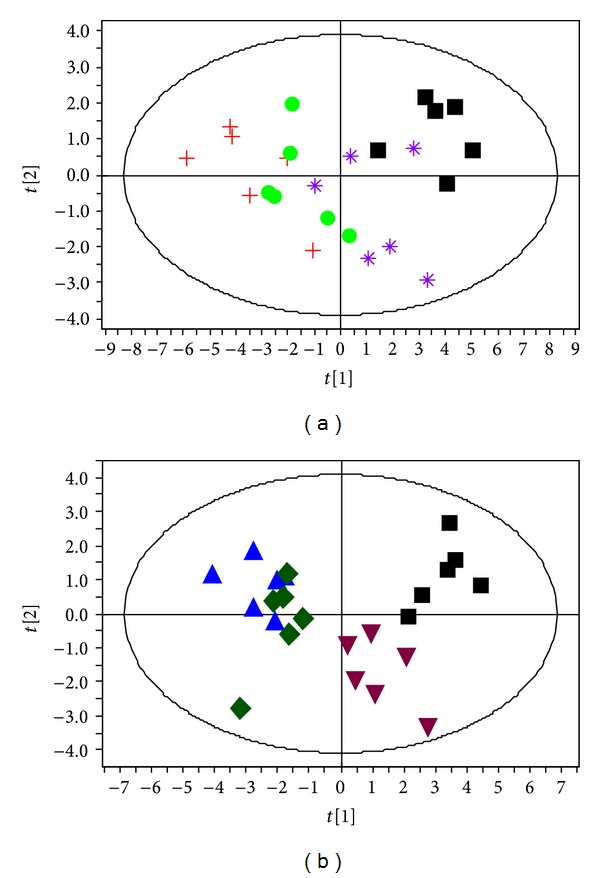
PCA scores plots indicating discrimination in therapeutic effects of different formulas. The two plots were generated based on serum levels of seventeen and fifteen significantly changed metabolites associated with SH (a) and SC (b), respectively. (black square) Control group, (red plus) SH model group, (blue triangle) SC model group, (purple asterisk) SH + ZJW treatment group, (light green circle) SH + LZW treatment group, (dark green diamond) SC + ZJW treatment group, and (maroon inverted triangle) SC + LZW treatment group.

As the 17 and 15 potential biomarkers associated with SH and SC, respectively, have been found, it is reasonable to take them as the potential drug targets for further investigating the therapeutic effects of the corresponding formula ZJW and LZW in TCM. Therefore, two PCA models, employing the levels of the 17 and 15 biomarkers as variables, respectively, were constructed to determine the effectiveness of different formulas on different TCM syndromes (Figure 6). The first and second principal components (*t*[1] and *t*[2]) calculated accounted for a total of 68.6% and 58.9% of variance for Figures 6(a) and 6(b), respectively. As shown in Figure 6(a), the SH + ZJW treatment group was closer to the control group and the SH + LZW treatment group was located near the SH model group, suggesting that the metabolic perturbation induced by SH could only be alleviated by administration of ZJW. Scores plot of Figure 6(b) showed two distinct clusters: the SC + ZJW treatment group and SC model group in the left side, the SC + LZW treatment group and control group in the right side, and the conclusion could be made that only LZW could reverse the pathological process of SC. The relative levels of the potential biomarkers in SH + ZJW treatment group and SC + LZW treatment group were illustrated in the heat map (Figure 4). Among the 17 potential biomarkers associated with SH, 13 including isoleucine, leucine, methionine, choline, betaine, phosphocholine, pyruvate, lactate, alanine, glutamate, VLDL/LDL –CH_2_–, lipid =CHCH_2_CH=, and lipid CH=CH were significantly reversed by ZJW compared to SH model group. Meanwhile, among the 15 potential biomarkers associated with SC, 10 including methionine, lysine, valine, phosphocholine, *β*-glucose, *α*-glucose, pyruvate, lactate, alanine, and glutamate were significantly reversed by LZW in comparison to SC model group. In brief, ZJW and LZW had therapeutic effects on the corresponding SH and SC through partially restoring balance to the perturbed metabolic pathways. However, no statistically significant change was observed in the relative levels of all the potential biomarkers between SH and SH + LZW groups, as well as SC and SC + ZJW groups. This confirmed that it was associated with the preventive effects but not from the TCM formulas themselves that the metabolic state of SH + ZJW and SC + LZW treatment groups was close to that of control group, because the complex TCM prescriptions might be a source to induce metabolic changes in animals.

## 4. Conclusions

In this study, differentiation between SH and SC could easily be made based on serum metabolic profiles, although no obvious difference was observed in macroscopic and histopathological examinations. Metabonomic studies enabled a more sensitive, rigorous, and comprehensive characterization of pathophysiological changes caused by SH and SC. In addition, effectiveness of ZJW and LZW in the treatment of the corresponding SH and SC was verified by using the identified potential biomarkers as screening indexes. Results from this investigation offered novel insights into understanding and explanation of “*Fang Zheng Dui Ying*” principle at metabolic and molecular levels.

## Supplementary Material

Figure 1S showed the representative ^1^H NMR spectra of rat serum of four treatment groups. PCA scores plots derived from ^1^H NMR spectra of rat serum samples in control and model groups were displayed in Figure 2S.

## Figures and Tables

**Table 1:** Gross evaluation of gastric mucosal lesions.

Animal group	Ulcer area (mm^2^)	Inhibition (%)
SH	713.45 ± 88.29	—
SH + ZJW	34.22 ± 2.19^c^	95.20^a^
SH + LZW	580.93 ± 74.46	18.57^a^
SC	702.13 ± 93.67	—
SC + ZJW	535.80 ± 136.11	23.69^b^
SC + LZW	49.20 ± 5.45^d^	92.99^b^

Values were expressed as means ± S.D. (*n* = 6). ^a^Inhibition was calculated from SH model group while ^b^inhibition was calculated from SC model group.

^c^ *P* < 0.05 compared to SH model group and ^d^ *P* < 0.05 compared to SC model group.

**Table 2:** Identified differential metabolites in rat serum.

No.	Metabolite	SH versus control			SC versus control			SH versus SC		
		VIP^a^	FC^b^	*P* ^c^	VIP^a^	FC^b^	*P* ^c^	VIP^a^	FC^b^	*P* ^c^
1	Isoleucine	1.62	0.74	2.82 × 10^−6^	1.50	0.78	3.47 × 10^−6^	/	0.94	—
2	Leucine	1.36	0.83	2.76 × 10^−2^	1.57	0.82	4.75 × 10^−3^	/	1.01	—
3	Methionine	3.67	0.76	2.17 × 10^−4^	3.04	0.83	1.22 × 10^−3^	1.38	0.91	—
4	Lysine	1.28	0.63	1.09 × 10^−3^	1.78	0.83	1.80 × 10^−2^	/	0.76	—
5	Valine	/	0.84	—	1.54	0.75	1.45 × 10^−3^	/	1.12	—
6	Choline	3.14	0.68	5.33 × 10^−7^	/	0.94	—	2.31	0.72	1.84 × 10^−5^
7	Betaine	4.16	0.70	2.66 × 10^−4^	1.69	0.90	—	2.56	0.78	7.80 × 10^−3^
8	Creatine	2.16	0.80	3.19 × 10^−3^	/	0.93	—	1.30	0.86	2.88 × 10^−2^
9	Phosphocholine	2.99	0.70	1.58 × 10^−3^	2.43	0.79	7.04 × 10^−3^	1.01	0.89	—
10	Trimethylamine	1.18	0.51	1.89 × 10^−5^	1.36	0.68	4.13 × 10^−3^	1.21	0.74	1.77 × 10^−2^
11	*β*-Glucose	/	1.03	—	2.12	0.54	5.21 × 10^−4^	1.56	1.91	2.20 × 10^−2^
12	*α*-Glucose	1.13	1.29	—	1.19	0.76	3.90 × 10^−2^	1.54	1.70	2.41 × 10^−2^
13	Pyruvate	2.06	0.74	5.91 × 10^−3^	2.78	1.49	1.00 × 10^−2^	3.06	0.50	6.41 × 10^−4^
14	Lactate	2.67	0.79	3.90 × 10^−2^	5.01	1.45	3.18 × 10^−5^	4.90	0.54	3.10 × 10^−5^
15	Alanine	2.36	0.71	2.55 × 10^−4^	1.58	1.17	1.84 × 10^−2^	2.55	0.60	3.87 × 10^−6^
16	Glutamate	1.72	0.70	1.37 × 10^−5^	1.33	1.19	2.88 × 10^−4^	1.86	0.59	6.45 × 10^−8^
17	VLDL/LDL –CH_2_–	4.39	1.68	1.07 × 10^−2^	/	0.99	—	3.45	1.69	1.05 × 10^−2^
18	Lipid =CHCH_2_CH=	1.34	1.46	4.07 × 10^−2^	/	0.84	—	1.31	1.74	8.89 × 10^−3^
19	Lipid CH=CH	2.52	1.52	2.31 × 10^−2^	/	0.94	—	2.11	1.61	1.51 × 10^−2^
20	Acetoacetate	1.72	0.69	8.12 × 10^−3^	2.52	0.49	6.42 × 10^−5^	1.18	1.43	5.94 × 10^−3^
21	Acetone	1.09	1.15	—	1.68	0.76	2.60 × 10^−2^	1.91	1.50	3.68 × 10^−3^

^a^VIP: variable importance in the projection; metabolites with VIP values ≥1.0 were considered significant; ^b^Fold change (FC) was calculated as the ratio of the mean metabolite levels between two groups; ^c^ *P* values were calculated from Student's *t*-test (equal variances assumed) or Mann-Whitney *U*-test (equal variances not assumed) with a threshold of 0.05. The symbol “—” represents statistically nonsignificant values (*P* > 0.05) and “/” denotes VIP values <1.0.
