# Supplementary material for: NMR-Based Metabonomic Studies on Stomach Heat and Cold Syndromes and Intervention Effects of the Corresponding Formulas
Source: Evid Based Complement Alternat Med. 2014 Feb 20;2014:528396. doi: 10.1155/2014/528396 (PMC3950656; doi:10.1155/2014/528396)
Supplement: Supplementary file 1 — Figure 1S showed the representative 1H NMR spectra of rat serum of four treatment groups. PCA scores plots derived from 1H NMR spectra of rat serum samples in control and model groups were displayed in Figure 2S. [file 528396.f1.pdf]

NMR-based metabonomic studies on stomach heat and cold syndromes and intervention effects of the corresponding formulas

Zhongjie Zou, Bin Han, Mengjuan Gong, Shumei Wang, and Shengwang Liang

School of Traditional Chinese Medicine, Guangdong Pharmaceutical University, Guangzhou 510006, China.

Correspondence should be addressed to Zhongjie Zou; zouzhongjie@139.com

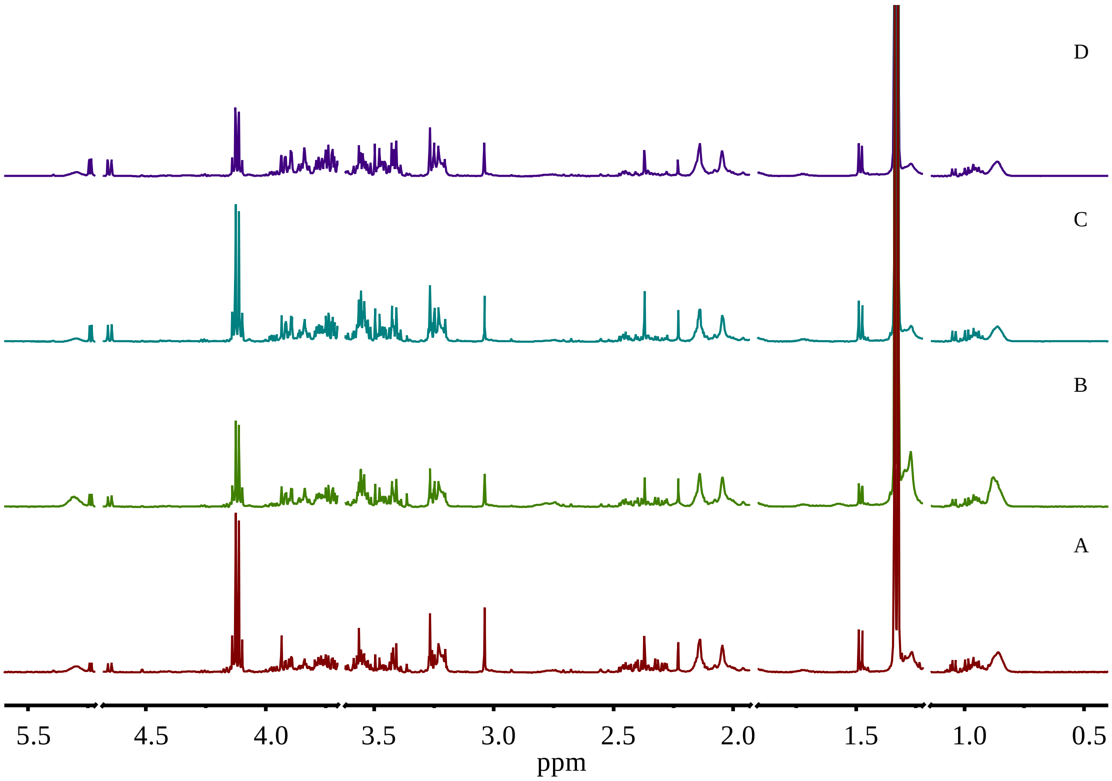

Figure 1S Representative ^{1}H NMR spectra of rat serum. (A) SH+ZJW treatment group, (B) SH+LZW treatment group, (C) SC+ZJW treatment group, (D) SC+LZW treatment group.

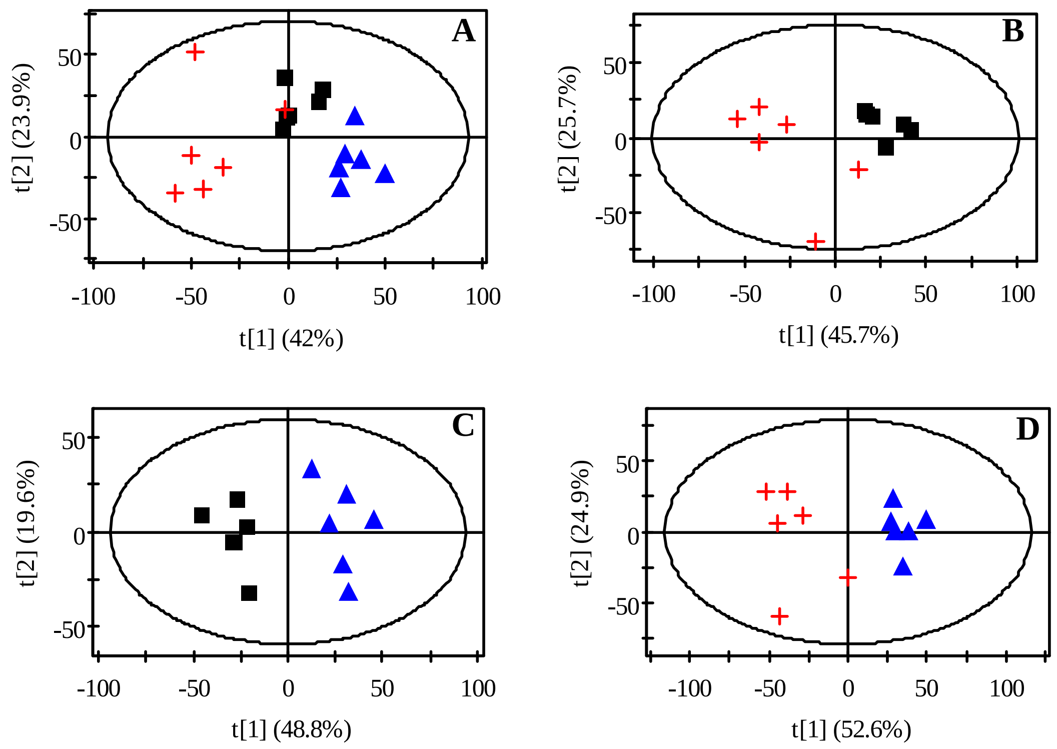

Figure 2S PCA scores plots derived from ^{1}H NMR spectra of rat serum samples. (■) Control group, (+) SH model group, (▲)SC model group. No outlier was observed. The four scores plots showed obvious separation between SH model group and control group, SC model group and control group, as well as SH and SC model groups.
